# Low serum adrenic acid levels in infants and subsequent food-induced anaphylaxis

**DOI:** 10.1016/j.jacig.2024.100291

**Published:** 2024-06-12

**Authors:** Mitsuyoshi Urashima, Ayu Kasamatsu, Hiroshi Tachimoto

**Affiliations:** a Division of Molecular Epidemiology, the Jikei University School of Medicine, Tokyo, Japan; b Department of Pediatrics, the Jikei University School of Medicine, Tokyo, Japan

**Keywords:** Anaphylaxis, food allergy, food-induced anaphylaxis, adrenic acid, AdA, docosatetraenoic acid (DTA), leukotriene B4, prostaglandin E2, infants

## Abstract

**Background:**

The dietary fat hypothesis links increases in allergic diseases to reduced consumption of n-3 polyunsaturated fatty acids from fish, for example, eicosapentaenoic acid, and increased intake of n-6 polyunsaturated fatty acids from vegetable oils, for example, arachidonic acid.

**Objective:**

Building upon the “fat hypothesis,” we sought to investigate the association between 24 types of serum fatty acid levels in infants and the risk of subsequent food-induced anaphylaxis (FIA) by age 2 years as the primary outcome.

**Methods:**

This study was conducted as a prespecified supplemental analysis within the ABC randomized clinical trial. We measured levels of 24 fatty acids in residual serum samples collected from 268 infants at age 5 to 6 months using gas chromatography-mass spectrometry.

**Results:**

Among the 258 infants, 58 exhibited immediate-type food allergies, whereas 200 showed no food allergy. Of the 58 infants, 12 were diagnosed with FIA, whereas the remaining 46 had nonanaphylactic food allergy. Unexpectedly, among the 24 fatty acids, only adrenic acid, also known as docosatetraenoic acid, which is one of the n-6 polyunsaturated fatty acids, showed significantly lower levels in infants with FIA (median [interquartile range] (wt.%), 0.16 [0.14-0.17]), compared with those with no food allergy (0.19 [0.17-0.21]) (*P* = .0007). In contrast, adrenic acid levels in infants with nonanaphylactic food allergy were 0.19 [0.16-0.21] (wt.%), which did not differ significantly from those in infants with no food allergy (*P* = .69).

**Conclusions:**

This study generated a hypothesis suggesting that infants with low serum adrenic acid levels might be at greater risk of subsequent FIA. This unexpected result warrants further investigation.

## Introduction

Food-induced anaphylaxis (FIA) can be life-threatening, especially in children and adolescents.^1^ Prevention primarily relies on allergen avoidance.^2^ Hence, continuing research to enable FIA prevention is of utmost importance. The dietary fat hypothesis suggests that the increase in allergic diseases in recent decades is paralleled by a decrease in consumption of oily fish that contain n-3 polyunsaturated fatty acids (PUFAs), such as eicosapentaenoic acid (EPA), docosapentaenoic acid (DPA), and docosahexaenoic acid (DHA), along with an increase in consumption of margarine and vegetable oils containing n-6 PUFAs, such as arachidonic acid (AA).^3^^,^^4^ Indeed, AA is converted to substances such as prostaglandin E2, which reportedly influences IgE production,^5^ and leukotriene B4 (LTB4), known for its leukocyte-activating properties.^6^ Conversely, EPA and DHA have been shown to inhibit prostaglandin E2 synthesis.^7^ Consequently, a high EPA/AA ratio is regarded as a promising indicator of reduced inflammation.^8^

We previously conducted the ABC trial (UMIN000011577), in which newborns were randomly assigned immediately after birth to either a breast-feeding (BF) with or without an amino acid–based elemental formula (BF/EF) group, or a BF supplemented with a small amount of cow’s milk formula (BF + CMF) group, for at least the first 3 days after birth. None of the infants was receiving EF at the time of blood sampling at age 5 to 6 months or later. The trial concluded that clinical food allergies and asthma or recurrent wheezing might be preventable by avoiding CMF supplementation for at least the first 3 days of life.^9^^,^^10^ Building on the “dietary fat hypothesis,” we aimed to investigate the association between serum levels of 24 types of fatty acids in infants and their subsequent risk of FIA as a prespecified supplemental analysis within the ABC trial.

## Results and discussion

**Fig 1:**
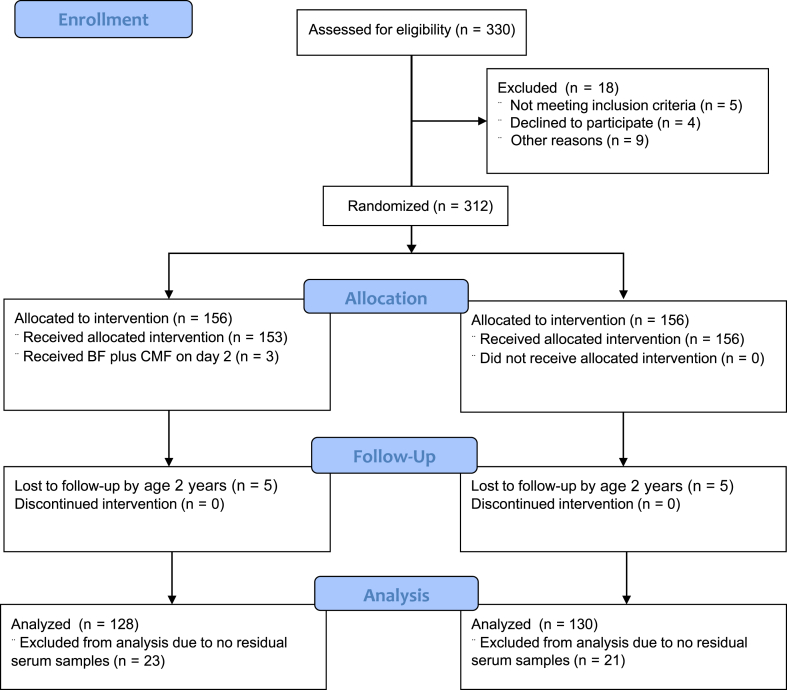
Flow diagram of study participation.

The ABC trial enrolled 312 pregnant women and their infants who were randomly assigned to either the BF/EF group or the BF + CMF group from the first day of the infant’s life in a 1:1 ratio (Fig 1). Blood samples were collected from 309 of 312 infants at age 5 to 6 months. Of these, 268 residual serum samples were available for measuring 24 different fatty acids. However, because of 10 infants (5 in each group) being lost to follow-up by their second birthday, 258 infants were finally analyzed for the primary outcome of FIA by age 2 years, which was determined on the basis of presence of at least 2 symptoms from different organ systems, for example, hives and asthmatic signs, and increased levels of suspected food-specific IgE. Presence of symptoms of only 1 organ system, for example, hives, was defined as nonanaphylactic food allergy (NAFA). The participants’ characteristics were similar between the BF/EF and BF + CMF groups (see Table E1 in this article’s Online Repository at www.jaci-global.org). Furthermore, the levels of the 24 different serum fatty acids were not significantly different between the 2 groups.

Among the 258 infants analyzed, immediate-type food allergies were observed in 58 infants (22.5%), whereas 200 (77.5%) remained unaffected, and were defined as no food allergy (NFA) (category 0). Of the 58 infants with food allergies, 12 (4.7%) were diagnosed with FIA (category 3) (wheat, 3; milk, 2; egg, 2; sesame, 2; walnuts, 2; soy, 1), whereas the remaining 46 had NAFA. However, 37 of the 58 (14.3%) infants had outgrown their allergies (category 1), whereas, in 9 (3.5%) infants, food allergies persisted beyond their second birthday (category 2).

We measured levels of the 24 fatty acids in residual serum samples collected from 268 infants at age 5 to 6 months using gas chromatography-mass spectrometry and expressed them as both the percentage by weight of each fatty acid relative to the total weight of the fat content (wt.%) and level of μg/mL in the serum samples. We performed Kruskal-Wallis rank tests for 3-group comparisons, followed by the Mann-Whitney test with Bonferroni correction for comparisons between 2 groups (Table I). Unexpectedly, among the 24 fatty acids, only adrenic acid (AdA), also known as docosatetraenoic acid (DTA), which is an n-6 PUFA, had significantly lower levels in infants with FIA (median [interquartile range] (wt.%) of 0.16 [0.14-0.17]), compared with those with NFA (0.19 [0.17-0.21]) (*P* = .0007). Even following adjustment for intervention and feeding patterns, AdA levels remained significantly associated with the risk of FIA (*P* = .005). In contrast, AdA levels in infants with NAFA were 0.19 [0.16-0.21] (wt.%), which did not significantly differ from those in infants with NFA (*P* = .69). In addition, the EPA/AA ratio in infants with NFA was 0.11 ([0.08-0.16]), which was significantly lower than in those with FIA (0.19 [0.11-0.26], *P* = .008), but equivalent to that in those with NAFA (0.11 [0.09-0.19], *P* = .54). Conversely, there were no differences in EPA, AA, and DPA levels between infants with NFA and FIA.

**Table I:** Association between serum fatty acid levels∗ and EPA/AA ratios in infants and FIA in toddlers

	Fatty acid	NFA (n = 200)†	FIA (n = 12)†	NAFA (n = 46)†	Kruskal-Wallis rank test‡*P* value, NFA vs FIA vs NAFA	Mann-Whitney test *P* value§	
						NFA vs FIA	NFA vs NAFA
1	Lauric acid SFA 12:0	0.79 (0.51-1.10) 26.5 (16.1-43.7)	0.86 (0.59-1.21) 26.6 (18.5-49.4)	0.66 (0.45-1.14) 20.7 (13.5-39.6)	.37	.73	.18
2	Myristic acid SFA 14:0	1.31 (1.05-1.74) 45.5 (31.2-70.5)	1.60 (1.14-2.08) 52.8 (34.9-89.0)	1.30 (1.01-1.74) 43.2 (30.9-58.7)	.42	.26	.61
3	Myristoleic acid MUFA 14:1 n-5	0.04 (0.03-0.05) 1.35 (0.90-2.10)	0.04 (0.04-0.06) 1.35 (1.00-2.35)	0.04 (0.03-0.05) 1.40 (1.00-1.80)	.98	.87	.90
4	Palmitic acid SFA 16:0	22.3 (21.6-22.9) 780 (652-952)	21.9 (21.5-22.6) 746 (673-945)	22.5 (21.5-23.0) 750 (648-880)	.45	.21	.99
5	Palmitoleic acid MUFA 16:1 n-7	1.24 (1.10-1.41) 42.7 (35.3-55.7)	1.20 (1.00-1.37) 39.5 (32.4-56.5)	1.32 (1.15-1.48) 48.0 (34.3-54.3)	.23	.47	.14
6	Stearic acid SFA 18:0	8.67 (8.17-9.13) 303 (264-347)	8.52 (8.17-9.33) 295 (272-371)	8.66 (8.31-9.09) 284 (256-350)	.72	.86	.43
7	Oleic acid MUFA 18:1 n-9	21.1 (19.6-23.2) 722 (594-957)	21.1 (19.1-22.6) 688 (657-829)	21.0 (19.0-22.8) 686 (545-893)	.53	.69	.28
8	Linoleic acid 6-n PUFA 18:2 n-6	27.1 (24.8-28.8) 957 (818-1091)	26.7 (24.5-29.5) 1001 (920-1054)	26.7 (25.2-28.4) 893 (798-1038)	.92	.68	.87
9	γ-Linolenic acid PUFA 18:3 n-6	0.13 (0.11-0.16) 4.80 (3.80-5.85)	0.13 (0.11-0.15) 4.50 (4.25-5.00)	0.13 (0.11-0.14) 4.20 (3.80-5.40)	.61	.51	.42
10	α-Linolenic acid PUFA 18:3 n-3	0.66 (0.56-0.78) 22.6 (18.1-31.4)	0.78 (0.57-0.99) 28.7 (18.9-35.6)	0.58 (0.48-0.74) 18.5 (14.2-29.6)	.04	.16	.03
11	AdA SFA 20:0	0.36 (0.34-0.40) 13.0 (11.7-14.6)	0.34 (0.32-0.38) 12.6 (11.0-14.5)	0.39 (0.34-0.42) 12.8 (11.5-14.6)	.06	.10	.12
12	Eicosenoic acid MUFA 20:1 n-9	0.19 (0.16-0.23) 6.60 (5.00-9.20)	0.21 (0.17-0.22) 6.75 (5.35-9.40)	0.19 (0.15-0.23) 6.50 (4.50-9.10)	.57	.48	.47
13	Eicosadienoic acid 6-n PUFA 20:2 n-6	0.26 (0.24-0.27) 8.90 (7.70-10.5)	0.26 (0.23-0.29) 9.50 (7.75-11.0)	0.26 (0.24-0.29) 8.50 (7.10-10.7)	.57	.86	.30
14	5,8,11-Eicosatrienoic acid PUFA 20:3 n-9	0.06 (0.05-0.07) 2.10 (1.70-2.65)	0.05 (0.04-0.06) 1.75 (1.50-2.35)	0.06 (0.05-0.07) 2.10 (1.60-2.40)	.12	.04	.68
15	Dihomo-γ-linolenic acid PUFA 20:3 n-6	0.82 (0.71-1.00) 29.8 (23.7-36.9)	0.86 (0.78-1.01) 31.7 (28.0-34.3)	0.91 (0.77-1.02) 31.3 (26.4-36.8)	.18	.67	.07
16	AA PUFA 20:4 n-6	6.15 (5.40-7.15) 215 (192-259)	5.69 (4.95-6.87) 215 (177-243)	6.34 (5.67-7.09) 227 (190-244)	.46	.23	.88
17	EPA PUFA 20:5 n-3	0.63 (0.49-1.01) 23.8 (17.0-37.3)	1.03 (0.58-1.85) 32.2 (21.4-66.3)	0.69 (0.52-1.16) 23.9 (17.3-43.9)	.15	.06	.48
18	Behenic acid SFA 22:0	0.66 (0.56-0.74) 23.2 (20.6-25.6)	0.62 (0.52-0.69) 21.8 (212-23.9)	0.67 (0.59-0.77) 23.3 (20.9-25.3)	.14	.16	.21
19	Erucic acid MUFA 22:1 n-9	0.05 (0.04-0.05) 1.60 (1.20-1.90)	0.05 (0.04-0.05) 1.70 (1.50-1.95)	0.05 (0.04-0.05) 1.55 (1.20-2.00)	.81	.59	.68
20	AdA 6-n PUFA 22:4 n-6	0.19 (0.17-0.21) 6.60 (5.80-7.60)	0.16 (0.14-0.17) 5.65 (5.00-6.00)	0.19 (0.16-0.21) 6.35 (5.50-7.60)	**.003**	**.0007**||	.69||
21	DPA PUFA 22:5 n-3	0.42 (0.36-0.52) 15.4 (12.1-19.0)	0.49 (0.44-0.54) 18.5 (15.2-20.0)	0.49 (0.39-0.54) 16.5 (14.1-18.6)	.15	.22	.11
22	Lignoceric acid SFA 24:0	0.56 (0.48-0.65) 19.5 (17.2-22.3)	0.51 (0.45-0.60) 18.8 (17.8-19.5)	0.59 (0.52-0.67) 20.0 (18.1-22.0)	.10	.16	.14
23	DHA PUFA 22:6 n-3	4.13 (3.65-4.86) 147 (125-177)	4.21 (3.82-5.31) 158 (128-193)	4.42 (3.71-5.06) 151 (134-179)	.40	.52	.21
24	Nervonic acid MUFA 24:1 n-9	1.17 (0.95-1.40) 41.4 (34.7-46.5)	1.28 (1.03-1.46) 44.9 (40.1-51.8)	1.24 (0.98-1.43) 42.4 (36.8-47.7)	.35	.46	.19
25	EPA/AA ratio	0.11 (0.08-0.16)	0.19 (0.11-0.26)	0.11 (0.09-0.19)	**.03**	**.008**||	.54||

*MUFA*, Monounsaturated fatty acid; *SFA*, saturated fatty acid.

∗ Serum levels of each fatty acid are presented as the median % (interquartile range) of both the percentage of serum fat weight (wt.%) and μg/mL.

† Weight % in total fat μg/mL in serum.

‡ The serum levels of fatty acids (wt%) were initially compared between 3 groups (NFA, FIA, and NAFA) using the Kruskal-Wallis rank test to identify significantly associated fatty acids (cutoff *P* value: .05). If the *P* value is statistically significant, it is shown in bold.

§ Subsequently, the Mann-Whitney test was used to compare levels of significant fatty acids between NFA, FIA, and NAFA groups. The Bonferroni correction was applied to mitigate type I errors, with the significance level set by dividing 0.05 by the total number of Mann-Whitney tests conducted. If the *P* value is statistically significant, it is shown in bold.

‖ In this case, because we conducted the Mann-Whitney tests 4 times, we considered *P* values less than .0125 to be statistically significant after adjusting for multiple comparisons (*α* = 0.05/4). If the *P* value is statistically significant, it is shown in bold.

**Fig 2:**
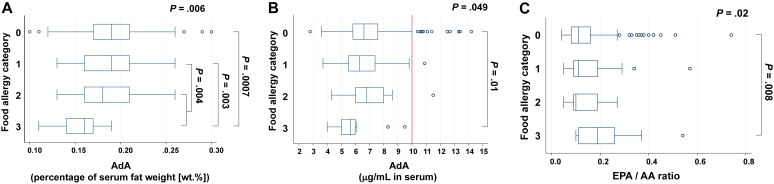
Serum AdA levels presented as a percentage of serum fat weight [wt. %] (**A**) and as the serum value in μg/mL (**B**), and EPA/AA ratios (**C**) across the 4 food allergy categories.

Next, we compared AdA levels across 4 food allergy categories. AdA levels (wt.%) significantly decreased with increase of the categories (*P* = .006) (Fig 2, *A*). In addition, AdA levels in infants with persistent food allergy at their second birthday, that is, category 2 plus category 3, were lower than in those who outgrew their immediate-type food allergy by their second birthday, that is, category 1 (*P* = .004). Comparison of serum AdA levels (μg/mL) among the 4 categories is shown in Fig 2, *B*. In addition, no FIA cases were observed when the serum AdA level exceeded 10 μg/mL. However, these findings also imply that lower serum AdA levels might not always lead to FIA. Conversely, EPA/AA ratios increased significantly with increase of the categories (*P* = .02) (Fig 2, *C*).

Unexpectedly, low AdA levels were found to be associated with an increased risk of FIA, despite AdA being one of the n-6 PUFAs that are generally considered proinflammatory.^11^ Previously, AdA levels in cord blood and breast milk were reported to be lower in infants at risk of atopic disease and allergic children than in infants not at risk and nonallergic, respectively.^12^^,^^13^ Recently, Brouwers et al^14^ showed that AdA inhibited the formation of the chemoattractant LTB4 in neutrophils and alleviated arthritis in a murine model. In this study, no FIA occurred when AdA levels exceeded 10 μg/mL. In addition to neutrophils, AdA is present at significant levels in membrane phospholipids of macrophages,^15^ and enhances macrophage phagocytosis.^14^ Hence, AdA might reduce the risk of FIA by inhibiting LTB4 production, at least in part. However, because food allergy involves more dendritic cells, lymphocytes, IgE, mast cells, and so on, the mechanism by which the risk of FIA increases when AdA is low necessitates further research.

**Fig 3:**
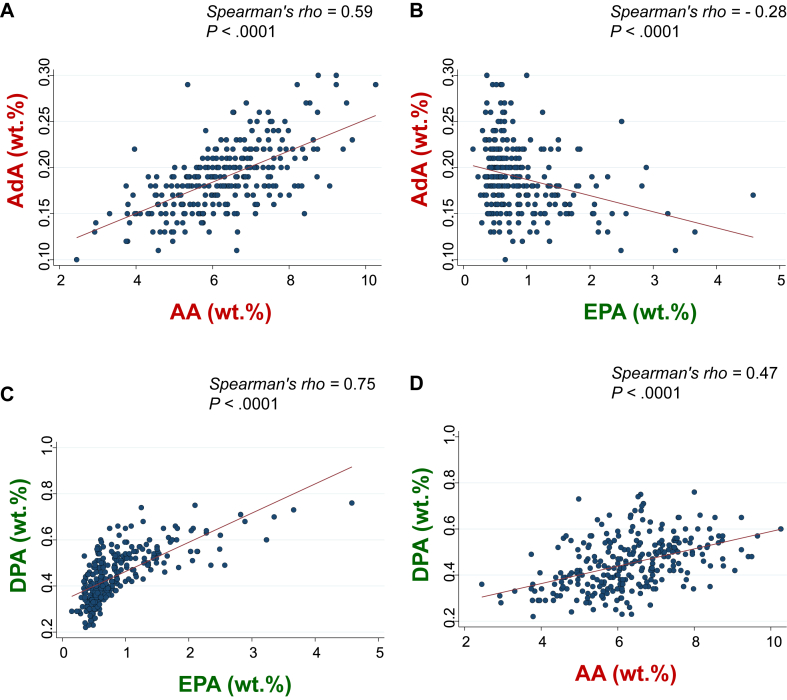
Association between AA and AdA (**A**), EPA and AdA (**B**), EPA and DPA (**C**), and AA and DPA (**D**).

**Fig 4:**
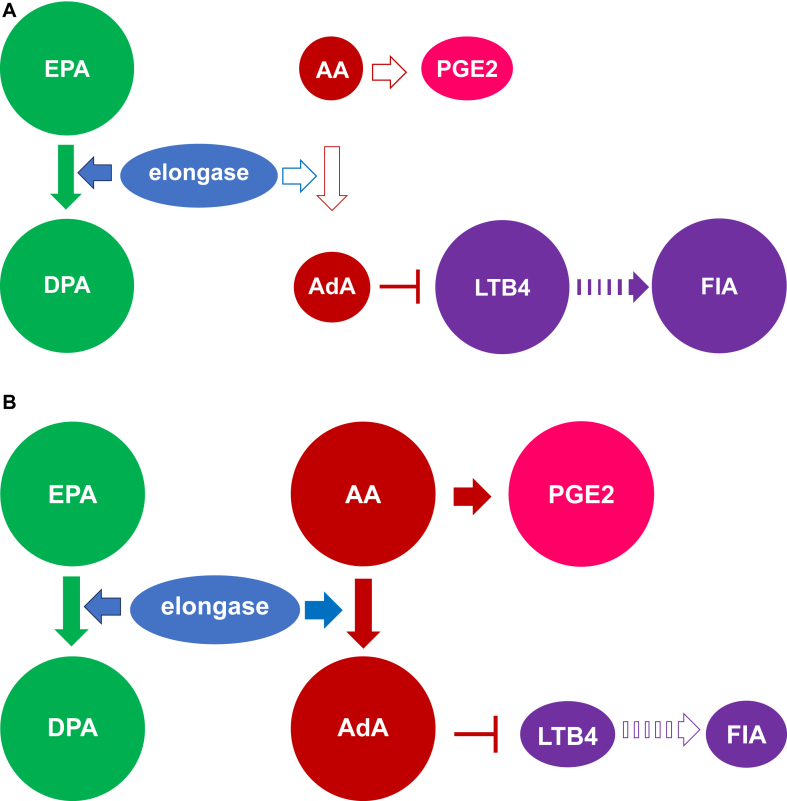
Hypothetical mechanisms of FIA triggered in infants with low AdA levels induced by high EPA/AA ratio (**A**), and not triggered in those with high AdA levels and a balanced EPA/AA ratio (**B**). *PGE2*, Prostaglandin E2. “→” means promotion, and “⊣” means inhibition. The solid arrows indicate strong metabolism, whereas the open arrows indicate weak metabolism. The dashed arrow between LTB4 and FIA indicates an unknown mechanism.

AA and EPA correlated positively and negatively, respectively, with AdA (Fig 3, *A* and *B*). In contrast, both EPA and AA correlated positively with DPA (Fig 3, *C* and *D*). However, there was no correlation between AdA and DPA. Because elongase is involved in both, metabolizing AA to AdA in the n-6 PUFA pathway and EPA to DPA in the n-3 pathway,^6^ AdA might decrease under conditions of high EPA/AA ratios (Fig 4, *A*) due to the lower availability of AA. In addition, the increased utilization of elongase in converting EPA to DPA could limit the conversion of AA to AdA, as seen by the negative association between EPA and AdA. Indeed, fish consumption and n-3 PUFA intake are reportedly associated with an increased risk of allergic diseases in Japan.^16^ Conversely, under conditions of low EPA/AA ratios (Fig 4, *B*), AdA might increase due to higher AA levels. However, the increased utilization of elongase in converting AA to AdA might not limit, but rather facilitate, the conversion of EPA to DPA, given the positive association between AA and DPA. These differential functions of elongase in metabolizing AA to AdA and EPA to DPA might explain why DPA did not exhibit an inverse correlation with AdA. Thus, consuming fish might increase EPA, which might indirectly decrease AdA, leading to an increased FIA risk. This, however, contradicts the observation that intake of n-3 PUFAs during late pregnancy reduces the risk of wheezing and asthma in infants.^17^ Conversely, previous meta-analyses showed that maternal n-3 PUFA supplementation during pregnancy, lactation, or infancy did not reduce the risk of food allergies in small children.^18^^,^^19^ However, the primary outcome in this study was FIA, and not asthma.

This study has several limitations. First, the results of this study were quite unexpected. In addition, scientific evidence supporting the results are scarce, suggesting that further validation studies are necessary. Second, although we suggested plausible mechanisms for the observed results, they remain speculative and unproven. Third, although EPA and DHA are abundant in fatty fish, specific food sources of AdA were not determined in our previous investigation.^20^ Fourth, only 12 infants developed FIA, which might have led to overestimation of the results. Fifth, only clinical criteria were used to distinguish children with FIA from those with NAFA. Moreover, this study did not take into account the molecular profiles of allergens, which could have caused a misclassification bias. Sixth, LTB4 levels in neutrophils were not measured in this study. Seventh, the study was conducted in central Tokyo, limiting its applicability in rural areas in Japan and other countries.

In conclusion, this study generated a hypothesis suggesting that infants with low serum AdA levels might be at greater risk of subsequent FIA. This unexpected result warrants further investigation.

**Key messages**

• Low serum AdA levels in infants are associated with a higher risk of subsequent FIA by age 2 years (primary outcome).
• Serum AdA levels in infants have no association with the risk of developing NAFA.
• None of the other 23 fatty acids analyzed, including AA and EPA, showed significant associations with the risk of developing any form of food allergy.

## Disclosure statement

This research was supported by the Practical Research Project for Allergic Diseases and Immunology of the 10.13039/100009619Japan Agency for Medical Research and Development, AMED (grant no. 15ek0410019h0101), and the 10.13039/501100007962Jikei University School of Medicine. The funders of the study played no role in study design, collection, analysis, and interpretation of data, and writing of the report, or put any restrictions regarding the submission of the report for publication.

Disclosure of potential conflict of interest: The authors declare that they have no relevant conflicts of interest.

## Supplementary data

Supplementary Table E1

## References

[1] Sampson H.A., Mendelson L., Rosen J.P. (1992). Fatal and near-fatal anaphylactic reactions to food in children and adolescents. N Engl J Med.

[2] Cianferoni A., Muraro A. (2012). Food-induced anaphylaxis. Immunol Allergy Clin North Am.

[3] Black P.N., Sharpe S. (1997). Dietary fat and asthma: is there a connection?. Eur Respir J.

[4] Lack G. (2008). Epidemiologic risks for food allergy. J Allergy Clin Immunol.

[5] Roper R.L., Brown D.M., Phipps R.P. (1995). Prostaglandin E2 promotes B lymphocyte Ig isotype switching to IgE. J Immunol.

[6] De Caterina R. (2011). n-3 fatty acids in cardiovascular disease. N Engl J Med.

[7] de Antueno R.J., de Bravo M.G., Toledo J., Mercuri O., De Tomás M.E. (1989). In vitro effect of eicosapentaenoic and docosahexaenoic acids on prostaglandin E2 synthesis in a human lung carcinoma. Biochem Int.

[8] Egalini F., Guardamagna O., Gaggero G., Varaldo E., Giannone B., Beccuti G. (2023). The effects of omega 3 and omega 6 fatty acids on glucose metabolism: an updated review. Nutrients.

[9] Urashima M., Mezawa H., Okuyama M., Urashima T., Hirano D., Gocho N. (2019). Primary prevention of cow’s milk sensitization and food allergy by avoiding supplementation with cow’s milk formula at birth: a randomized clinical trial. JAMA Pediatr.

[10] Tachimoto H., Imanari E., Mezawa H., Okuyama M., Urashima T., Hirano D. (2020). Effect of avoiding cow’s milk formula at birth on prevention of asthma or recurrent wheeze among young children: extended follow-up from the ABC randomized clinical trial. JAMA Netw Open.

[11] Sartorio M.U.A., Pendezza E., Coppola S., Paparo L., D’Auria E., Zuccotti G.V. (2021). Potential role of omega-3 polyunsaturated fatty acids in pediatric food allergy. Nutrients.

[12] Beck M., Zelczak G., Lentze M.J. (2000). Abnormal fatty acid composition in umbilical cord blood of infants at high risk of atopic disease. Acta Paediatr.

[13] Duchén K., Casas R., Fagerås-Böttcher M., Yu G., Björkstén B. (2000). Human milk polyunsaturated long-chain fatty acids and secretory immunoglobulin A antibodies and early childhood allergy. Pediatr Allergy Immunol.

[14] Brouwers H., Jónasdóttir H.S., Kuipers M.E., Kwekkeboom J.C., Auger J.L., Gonzalez-Torres M. (2020). Anti-inflammatory and proresolving effects of the omega-6 polyunsaturated fatty acid adrenic acid. J Immunol.

[15] Monge P., Garrido A., Rubio J.M., Magrioti V., Kokotos G., Balboa M.A. (2020). The contribution of cytosolic group IVA and calcium-independent group VIA phospholipase A2s to adrenic acid mobilization in murine macrophages. Biomolecules.

[16] Hamazaki K., Tsuchida A., Takamori A., Tanaka T., Ito M., Inadera H., Japan Environment and Children’s Study (JECS) Group (2019). Dietary intake of fish and ω-3 polyunsaturated fatty acids and physician-diagnosed allergy in Japanese population: the Japan Environment and Children’s Study. Nutrition.

[17] Bisgaard H., Stokholm J., Chawes B.L., Vissing N.H., Bjarnadóttir E., Schoos A.M. (2016). Fish oil-derived fatty acids in pregnancy and wheeze and asthma in offspring. N Engl J Med.

[18] Gunaratne A.W., Makrides M., Collins C.T. (2015). Maternal prenatal and/or postnatal n-3 long chain polyunsaturated fatty acids (LCPUFA) supplementation for preventing allergies in early childhood. Cochrane Database Syst Rev.

[19] Schindler T., Sinn J.K., Osborn D.A. (2016). Polyunsaturated fatty acid supplementation in infancy for the prevention of allergy. Cochrane Database Syst Rev.

[20] Kasamatsu A., Tachimoto H., Urashima M. (2023). Impact of maternal fish consumption on serum docosahexaenoic acid (DHA) levels in breastfed infants: a cross-sectional study of a randomized clinical trial in Japan. Nutrients.

