# Supplementary material for: Low serum adrenic acid levels in infants and subsequent food-induced anaphylaxis
Source: J Allergy Clin Immunol Glob. 2024 Jun 12;3(3):100291. doi: 10.1016/j.jacig.2024.100291 (PMC11277412; doi:10.1016/j.jacig.2024.100291)
Supplement: Supplementary Table E1 [file mmc1.docx]

**Online Repository**

**Title:**

**Low serum adrenic acid levels in infants and subsequent food-induced anaphylaxis**

**METHODS**

**Study population**

A total of 312 pregnant women and their infants were randomly assigned to either the breast feeding with or without elemental formula (BF/EF) group or the BF plus cow’s milk formula (CMF) group in a 1:1 ratio from the infant’s first day of life. Blood samples were collected from 309 infants when they were 5-6 months old, but only 268 residual serum samples were available for analysis of 24 different types of fatty acids. Additionally, 10 participants were lost to follow-up. Therefore, data from 258 participants were included in the analysis. There were no significant differences in the levels of the 24 kinds of fatty acids and eicosapentaenoic acid/arachidonic acid (EPA/AA) ratio between the BF/EF and BF plus CMF group.

**Study design**

This study was designed as a pre-specified study conducted as a supplemental analysis of the Atopy Induced by Breastfeeding or Cow’s Milk Formula (ABC) trial, which aimed to determine if avoiding or introducing CMF for at least the first three days of life might decrease the risk of sensitization to cow's milk protein and clinical food allergies. Enrollment began on October 1, 2013, and follow-up was completed on May 31, 2018, at a single university hospital in Tokyo, Japan. Written, informed consent was obtained from the parents of all the enrolled infants. The trial protocol was approved by the ethics committee of the Jikei University School of Medicine and the institutional review board of Jikei University Hospital (25-057(7192)). The trial was registered with the UMIN Clinical Trials Registry (UMIN000011577).

**Participants**

The inclusion criteria were infants at risk for atopy, due to at least one of their parents or siblings having current and/or past atopic diseases (e.g., asthma). The exclusion criteria consisted of parents who intended to exclusively provide breastfeeding or CMF before birth, or infants who were born at less than 36 weeks’ gestational age, had a birth weight of less than 2000 g, or had serious congenital anomalies (e.g., cleft palate).

**Infant formula and intervention**

Both CMF (Meiji Hohoemi^®^) and an amino acid-based elemental formula (EF, Meiji Elemental formula^®^) are marketed in Japan and were purchased from Meiji Holdings Co. Ltd. (Chuo-ku, Tokyo, Japan). Newborn infants were randomly assigned to one of two groups: [1] the breastfed or EF supplemented group (BF/EF), who were to avoid CMF for at least the first 3 days of life, but were allowed to receive amino acid–based EF when it was believed that the amount of breastfeeding was not enough, or [2] the breastfed and CMF supplemented group (BF+CMF), whose parents were asked to supplement breastfeeding with at least 5 mL of CMF from the first day or within the first 24 hours after delivery, and who received at least 40 mL/d of CMF after 1 month of age, which was continued until the first blood test at 5-6 months of age or before starting solid foods. If the mother in the BF/EF group added more than 150 mL/d of EF to breastfeeding for 3 consecutive days, the EF was switched to CMF after the fourth day. Hence, offspring allocated to the BF/EF group could avoid CMF for at least the first 3 days of life.

**Serum fatty acid measurements**

Blood was collected from the infants at 5-6 months of age, prior to the introduction of solid foods. In the ABC trial, 25-hydroxyvitamin D, total IgE, and antigen-specific IgE levels were measured. If sufficient residual serum samples were available, they were utilized to measure the composition of 24 serum fatty acids. The serum samples were stored at -80°C and sent to SRL Inc. in Hachioji, Tokyo, Japan for analysis by persons who were blinded to the clinical outcomes. The concentration of each fatty acid was measured by gas chromatography-mass spectrometry using a calibration curve method, and expressed as both the percentage of serum fat weight (wt.%) and the serum level in μg/mL.

**Outcome measures**

The primary outcome of this study was defined as food-induced anaphylaxis (FIA), which was determined based on the presence of at least two symptoms from different organ systems, such as the skin (e.g., urticaria), respiratory tract (e.g., wheezing), gastrointestinal tract (e.g., vomiting), or circulatory system (e.g., loss of consciousness). The anaphylaxis had to be triggered either by an oral food challenge (OFC) test administered by trained pediatricians, or by food ingestion in daily life, with symptoms appearing within minutes to a few hours. In addition, the primary outcome also included serum levels of suspected food-specific IgE levels equal to or greater than 0.35 UA/mL. The secondary outcomes of this study were the cumulative incidence of immediate types of food allergy without anaphylaxis, i.e., non-anaphylactic food allergy (NAFA), which were characterized by symptoms derived from one organ system, triggered either by the OFC test or by food ingestion in daily life, with symptoms appearing within minutes to a few hours, along with elevation of suspected food-specific IgE levels (≥0.35 UA/mL). The remaining infants were defined as having no food allergy (NFA).

**Oral food challenge test**

For infants who had food antigen–specific IgE-positive findings (≥0.35 UA/mL) at 5 months of age, the OFC test was performed at approximately 1 year of age at Jikei University Hospital. After blood examination at 5 months of age, if the infants had already taken the allergenic food without clinical signs of allergy, or, conversely, if the infants had already shown a strong allergic or anaphylactic reaction to the allergenic food, the OFC test was not performed.

**Food allergen immunotherapy**

Testing for non-anaphylactic food allergy (NAFA) began with exposure to small amounts of the food antigen, with observation for a reaction within a few hours. If infants reached the final step of an OFC test by the age of 2 years, they were classified as Category 1, indicating that they had outgrown the immediate-type food allergy by their 2nd birthday. Conversely, those unable to consume the food at this stage were classified as Category 2, signifying persistence of the immediate-type food allergy without FIA at their 2nd birthday.

**Statistical analysis**

Serum fatty acid levels (wt.%) were initially compared between three groups (NFA, FIA, and NAFA) using the Kruskal-Wallis rank test to identify significantly-associated fatty acids (cut-off p value: 0.05). Subsequently, the Mann-Whitney test was used to compare significantly different fatty acid levels between the NFA, FIA, and NAFA groups. Bonferroni correction was applied to mitigate type I errors, with the significance level set by dividing 0.05 by the total number of Mann-Whitney tests conducted. Spearman's rank correlation (rho) was employed to assess the strength and direction of the correlation. All data were analyzed using Stata, version 17.0 (Stata Corp).

**Table E1.** Participants’ characteristics

|  | **BF/EF**  n = 128 | **BF plus CMF**  n = 130 |
| --- | --- | --- |
| Family background |  |  |
| Mean maternal age (SD) - years | 35.0 (4.4) | 35.3 (4.2) |
| Mean paternal age (SD) - years | 37.0 (5.5) | 37.9 (6.9) |
| Current / previous atopic disease in mother |  |  |
| Bronchial asthma, no. (%) | 8 (6.3) / 22 (17.2) | 5 (3.9) / 17 (13.1) |
| Atopic dermatitis, no. (%) | 15 (11.7) / 29 (22.7) | 12 (9.2) / 26 (20.0) |
| Food allergy, no. (%) | 23 (18.0) / 17 (13.3) | 13 (10.0) / 13 (10.0) |
| Allergic rhinitis, no. (%) | 35 (27.3) / 15 (11.7) | 36 (27.7) / 13 (10.0) |
| Pollen allergy, no. (%) | 72 (56.3) / 11 (8.6) | 73 (56.2) / 10 (7.7) |
| Current / previous atopic disease in father |  |  |
| Bronchial asthma, no. (%) | 3 (2.3) / 17 (13.3) | 7 (5.4) / 21 (16.2) |
| Atopic dermatitis, no. (%) | 5 (3.9) / 11 (8.6) | 11 (8.5) / 19 (14.6) |
| Food allergy, no. (%) | 17 (13.3) / 2 (1.6) | 14 (10.8) / 7 (5.4) |
| Allergic rhinitis, no. (%) | 32 (25.0) / 10 (7.8) | 33 (25.4) / 15 (11.5) |
| Pollen allergy, no. (%) | 63 (49.2) / 7 (5.5) | 64 (49.2) / 3 (2.3) |
| Number of siblings, 0/1/2 | 125/2/1 | 125/4/1 |
| Perinatal status |  |  |
| Median gestational weeks (IQR) | 39 (38-39) | 39 (38-39) |
| Delivery |  |  |
| Planned Cesarean section, no. (%) | 15 (11.7) | 13 (10.0) |
| Emergency Cesarean section, no. (%) | 17 (13.3) | 20 (15.4) |
| Sex |  |  |
| Female, no. (%) | 69 (53.9) | 65 (50.0) |
| Apgar score at 5 minutes |  |  |
| 8 points | 2 (1.6) | 9 (7.1) |
| 9 points | 108 (87.8) | 106 (83.5) |
| 10 points | 13 (10.6) | 12 (9.5) |
| Mean placental weight (SD), g | 586.8 (105.2) | 566.7 (96.9) |
| Mean pH of cord blood (SD) | 7.28 (0.06) | 7.30 (0.08) |
| Mean anthropometric values at birth (SD) |  |  |
| Body weight, g | 2,994 (318.7) | 3,000 (316.4) |
| Body height, cm | 48.7 (1.7) | 48.8 (1.8) |
| Chest circumference, cm | 31.5 (1.4) | 31.6 (1.4) |
| Head circumference, cm | 33.9 (1.3) | 34.1 (1.2) |
